# Case Report: Response to Regional Melphalan *via* Limb Infusion and Systemic PD1 Blockade in Recurrent Myxofibrosarcoma: A Report of 2 Cases

**DOI:** 10.3389/fonc.2021.725484

**Published:** 2021-10-15

**Authors:** Edmund K. Bartlett, Sandra P. D’Angelo, Ciara M. Kelly, Robert H. Siegelbaum, Charles Fisher, Cristina R. Antonescu, Charlotte E. Ariyan

**Affiliations:** ^1^ Department of Surgery, Memorial Sloan Kettering Cancer Center, New York, NY, United States; ^2^ Department of Surgery, Weill Cornell Medical College, New York, NY, United States; ^3^ Department of Medicine, Memorial Sloan Kettering Cancer Center, New York, NY, United States; ^4^ Department of Medicine, Weill Cornell Medical College, New York, NY, United States; ^5^ Department of Radiology, Memorial Sloan Kettering Cancer Center, New York, NY, United States; ^6^ Department of Radiology, Weill Cornell Medical College, New York, NY, United States; ^7^ Department of Anesthesiology, Memorial Sloan Kettering Cancer Center, New York, NY, United States; ^8^ Department of Pathology, Memorial Sloan Kettering Cancer Center, New York, NY, United States

**Keywords:** Sarcoma, Immunotherapy, isolated limb infusion, undifferentiated pleomorphic sarcoma (UPS), myxofibrosarcoma

## Abstract

Treatment options for patients with advanced sarcoma remain limited. Promising responses to checkpoint inhibition have been observed, but responses to single-agent PD-1 inhibition are rare. We report on two patients with multiply recurrent myxofibrosarcoma treated with the combination of regionally administered melphalan (*via* isolated limb infusion) and pembrolizumab. Both patients had recurrent disease after multiple surgical resections and radiation. Analysis of primary tumors demonstrated microsatellite stable tumors with few mutations. After combination treatment, one patient had a significant partial response of 6 months duration, the second patient had a complete response of 2 years duration. Post treatment biopsies demonstrated immune infiltration into the tumor. These promising responses in patients with multiply recurrent myxofibrosarcoma have prompted the development of an investigator-initiated clinical trial to formally study the combination of regional melphalan and pembrolizumab in a systematic fashion (NCT04332874).

## Background

Prognosis of advanced sarcoma is poor, with a median survival of 10-15 months in the metastatic setting. Standard cytotoxic chemotherapy agents such as doxorubicin, ifosfamide, and dacarbazine result in objective responses in 10-25% of the patients, and complete responses are rare (1). The development of novel and effective therapies is desperately needed.

Immunotherapeutic strategies may be a promising approach to treating advanced sarcoma, but thus far the response rates are only 5-18% in studies of PD1 alone (2, 3). However, certain sarcoma subtypes may have greater response to immunotherapy than others. For example, analysis demonstrated increased response rated in undifferentiated pleomorphic sarcoma (UPS) group, a common sarcoma subtype, and prompted an expansion cohort that recently reported a 23% response (9/40 UPS patients) (4). A retrospective analysis of sarcoma patients treated with checkpoint inhibition at MD Anderson additionally identified alveolar soft part sarcoma (ASPS) as a histologic type responsive to pembrolizumab (5). As a result, pembrolizumab has recently been incorporated into the NCCN guidelines for treatment of UPS and ASPS (6). Additional combinatorial approaches to overcome the high rate of primary resistance are under investigation, with some promising responses reported (7, 8).

The mechanisms underlying the observed primary resistance to checkpoint inhibition are not well understood. Most sarcomas, however, have low tumor mutational burden and few tumor infiltrating lymphocytes (TILs); both factors known to associate with poor response to checkpoint inhibition (9). Despite this, when present in sarcomas, pretreatment PD-L1 expression and TIL are associated with a better prognosis, suggesting that local immune activation can overcome the low tumor mutational burden (10). Therefore, strategies to enhance inflammation within the tumor, as seen in other tumors such as melanoma, have therapeutic potential in sarcoma.

One strategy to increase immune infiltration into the tumor microenvironment is administration of chemotherapy. Chemotherapy may sensitize tumors to elimination by the immune system by induction of acute inflammation, inhibition of myeloid-derived suppressor cells, increasing the ratio of effector T cells to T-regulatory cells, and/or down-regulating checkpoint pathways *via* STAT6-mediated signaling (11). Local chemotherapy *via* isolated limb perfusion has been employed in sarcoma with some efficacy since the 1950s (12). Conceptually, this was developed to deliver high doses of chemotherapy to the tumor while minimizing the systemic toxicity associated with such doses. Previously, our institution completed a phase 2 study combining local chemotherapy with melphalan (*via* isolated limb infusion, ILI) and checkpoint inhibition (ipilimumab) for patients with melanoma (13). This combination of ILI and checkpoint inhibition resulted in a 1-year progression-free survival of 58% and a 3-month overall response rate of 85%. Of the two patients with metastatic disease in this trial, both developed a durable (at least 2 years) complete response of not only the regional disease but systemic metastases as well. The current study reports on two patients with recurrent myxofibrosarcoma who were treated off-protocol with a combination therapy of pembrolizumab and ILI and have demonstrated promising responses.

## Methods

The clinical treatment and follow-up for both patients is described below and occurred as part of standard clinical treatment. Both patients provided consent for study of specimens on an IRB-approved prospective tissue acquisition protocol. Both patients provided separate consent for the publication of their cases.

Immunohistochemistry was performed on formalin-fixed paraffin-embedded specimens. Stains utilized included CD3 (#NCL-L-CD3-565, Leica Biosystems), CD4 (#104R, Cell Marque), CD8 (#960-4460, Ventana), PD-L1 (#13684, Cell Signaling Technology), and CD163 (#760-4437, Ventana). Images are presented at 20x magnification. Individual mutation status, tumor mutational burden, and microsatellite status were assessed *via* MSK-IMPACT as previously described (14).

### Case 1

**Figure 1:**
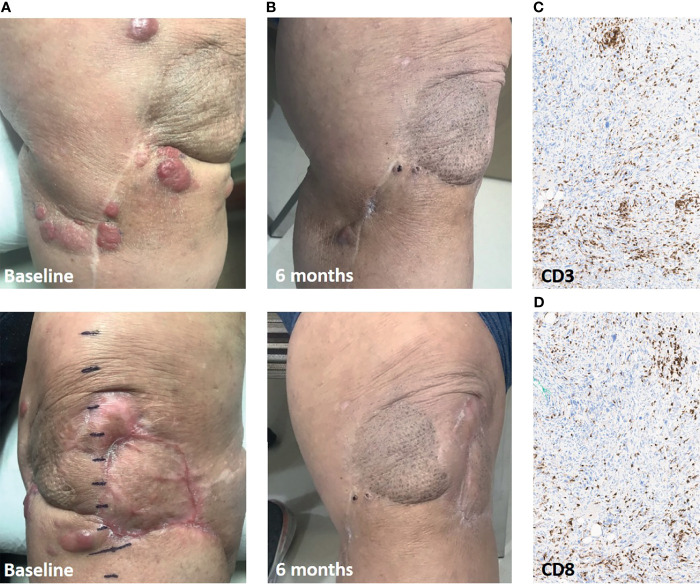
Response in Case 1 treated with ILI and pembrolizumab. **(A)**, Baseline clinical pictures of the numerous recurrent lesions. **(B)**, 6-month follow-up demonstrating significant partial response in the infused limb. **(C)**, CD3+ T cell infiltrate in leg soft-tissue biopsy of a responding lesion. **(D)**, CD8+ T cell infiltrate. Immunohistochemistry images are presented at 20x magnification.

The first patient is a 79 year-old female with a history of 5 prior surgical resections and radiation for a locally recurrent high grade myxofibrosarcoma of the lower extremity. Genomic analysis demonstrated alterations in tumor suppressor genes, including deletions in *TP53* (W146fs) and *CDKN2A*. The tumor was microsatellite stable, with a tumor mutational burden of 3.5 mutations per megabase. She was referred for amputation but opted for an ILI with concurrent pembrolizumab in an attempt to salvage the limb. At presentation she had numerous (at least 12) lesions in the vicinity of her prior operations but no evidence of distant disease (**Figure 1A**). She received a single dose of pembrolizumab followed by ILI within two weeks and was subsequently maintained on pembrolizumab post-infusion according to the standard dosing schedule. The patient had a partial response of 6 months duration (**Figure 1B**). All of the lesions that completely regressed continued with a complete response. At 5 months, she developed symptomatic hypothyroidism and pembrolizumab was held. Progression was noted in the lesions that had partially responded before pembrolizumab was resumed.

A biopsy of one of the responding lesions showed a brisk lymphocytic infiltrate (**Figures 1C, D** **)**. The patient went on to other limb salvage treatment when the remaining lesions grew off treatment. She remains alive with disease now 30 months post-ILI.

### Case 2

**Figure 2:**
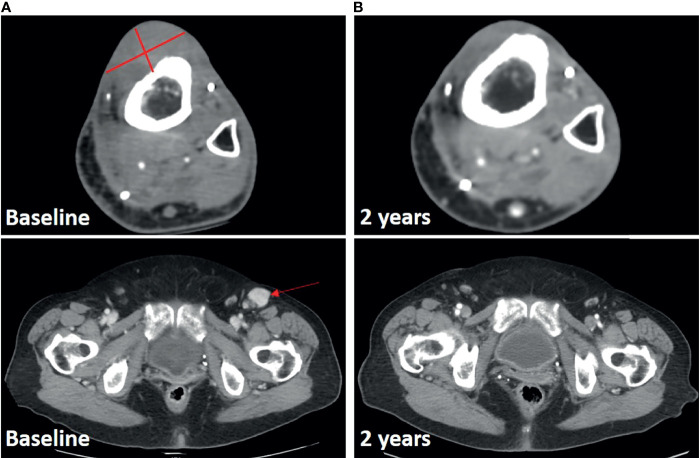
Response in Case 2 treated with ILI and pembrolizumab. **(A)**, Baseline CT scan with lesions noted. **(B)**, 2-year follow-up scan demonstrating ongoing response both in infused limb and at distant site.

The second patient is a 77 year-old female who had seven prior surgical resections as well as radiation for multiply recurrent high grade myxofibrosarcoma of the lower extremity. She presented one year after her 7^th^ resection with recurrent disease, a 4.1cm x 2.9cm mass overlying the anterior tibia, a possible adjacent soft-tissue tumor nodule, and metastatic disease in an inguinal lymph node (**Figure 2A**). Genomic analysis revealed a *TP53* (R342*) mutation and *ATRX* deletion. The tumor was microsatellite stable, with a tumor mutational burden of 0.9 mutations per megabase. She received a single dose of pembrolizumab followed by ILI a week later. She subsequently continued on pembrolizumab for one year. Her response to treatment was rapid with resolution of her palpable inguinal node and softening of her tibial lesion at her 1-month follow up. Both the lesion in the chemotherapy infusion field (tibia) and outside the infusion field (inguinal lymph node) demonstrate ongoing radiologic complete response at two years (**Figure 2B**).

**Figure 3:**
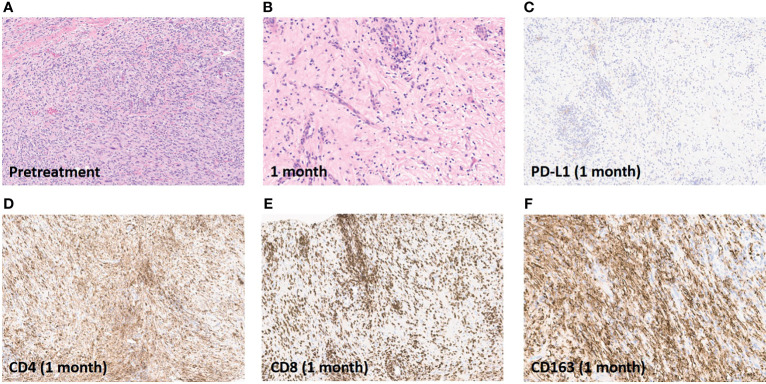
Pathologic and immunohistochemical assessment of response. **(A)**, Excision specimen of recurrent tumor one year prior to ILI. **(B)**, Tibial biopsy one month after ILI showing no viable tumor. **(C)**, Absence of PD-L1 expression post-ILI and PD-1 inhibition. **(D)**, Brisk CD4+ T cell infiltration in tibial biopsy one month after ILI. **(E)**, Brisk CD8+ T cell infiltration in tibial biopsy one month after ILI. **(F)**, Brisk CD163+ macrophage infiltration in tibial biopsy one month after ILI. Images are presented at 20x magnification.

Compared to the pathologic appearance of the excision specimen a year prior (**Figure 3A**), a tibial biopsy performed one month after ILI revealed >99% treatment effect (**Figure 3B**). PD-L1 expression was absent in the post-treatment biopsy, with extensive CD4+ and CD8+ T cell infiltrate, as well as CD163+ macrophage infiltrate (**Figures 3C–F**).

## Discussion

This report presents two cases demonstrating promising responses to PD-1 checkpoint inhibition when combined with regional chemotherapy in patients with multiply recurrent myxofibrosarcoma. To our knowledge, these are the first two cases of patients with sarcoma treated with this combination therapy. One response was durable for 6 months and the other is ongoing at two years. Responses were observed not only in the limb perfused with melphalan during the ILI, but also (in Case 2) in a regional lymph node which was outside of the field treated with chemotherapy.

Local chemotherapy *via* ILI alone has been reported to achieve response rates of ~50%, but the widespread adoption of this therapy has been limited by the short duration of response as well as the inability of the local therapy to impact on the development of distant metastases (15). In one large series of patients with sarcoma undergoing local chemotherapy, 40% developed distant recurrence during follow-up, with a median time to distant recurrence of 4.6 months (16). Thus our aim in combining regional chemotherapy with pembrolizumab is not just to enhance local control, but to sensitize these tumors to checkpoint inhibition and thereby potentiate the duration of response as well as treatment of systemic disease.

The highly inflamed tumor microenvironment observed in the post-treatment biopsies by immunohistochemistry in the two presented cases is consistent with the pro-inflammatory effects observed in patients treated with systemic chemotherapy in other tumor types (11, 13). Specifically in melanoma, *in vitro* melphalan treatment of B16 melanoma cells has been found to increase expression of MHC class I and PD-L1 (13). In a B16OVA mouse model of melanoma, the combination of melphalan and checkpoint blockade (anti-CTLA-4) improved survival and resulted in long term anti-tumor immunity in a majority of mice. Tumors treated with both therapies had fewer T-regulatory (Foxp3+) cells and an increased CD8/Treg ratio (13). Furthermore, melphalan exposure *via* isolated limb perfusion is associated with increased IL-1B, IL-6, and IL-8 within patient tumor samples (17). The gene expression pattern after ILI with melphalan demonstrates upregulation of proinflammatory transcripts (TREM1, CCL3, CCL3L1, TNFRSF10C), costimulatory ligands (CD80, CD86, ICOS), and MHC class I and II genes (13). When ILI was followed by checkpoint inhibition (ipilimumab) for patients with melanoma, increased expression of cytotoxic T-cell-effector transcripts (GZMA, GZMH, IFNγ, PRF1) was observed (13). Regional delivery of chemotherapy has particular theoretic appeal as a pro-inflammatory therapy. Regional therapy is tolerated at substantially higher doses than systemic therapy, potentially inducing greater inflammation, and the myelosuppressive effects of many systemic agents are avoided when therapy is delivered regionally.

It should be noted that the inflammation induced by limb infusion is not necessarily entirely anti-tumorigenic, M2-polarized macrophages (CD163+) may represent pro-tumorigenic inflammation (18). Macrophage infiltrate is frequently observed across sarcoma histologic types (10). In select subtypes, tumor-associated macrophages do appear to have a pro-tumorigenic role and have even recently been successfully targeted therapeutically in sarcoma models and in a Phase III trial for the treatment of tenosynovial giant-cell tumor (19, 20). The clinical responses observed in the two cases presented might suggest that the anti-tumorigenic response is dominant. However, if extensive CD163 infiltration is consistently observed in future patients, targeting this cell population may be a rational strategy to further improve responses in the future.

The tumor mutational burden in these two cases is consistent with the low average observed in patients with sarcoma (1.1 mutations/megabase observed among all sarcoma patients assessed with MSK-IMPACT, unpublished data). Unfortunately, as these patients were not treated on a clinical trial, the baseline immune infiltrate in these two cases immediately pre-treatment was not available for a pre-/post-treatment comparison, which limits the ability to directly attribute the observed inflammatory tumor microenvironment to the combination of local chemotherapy and checkpoint blockade. However, in light of the preclinical data, the results of a similar approach in melanoma, and these two promising responses, we have subsequently opened a clinical trial of ILI and pembrolizumab to formally study this combination in patients with sarcoma (NCT04332874).

## Data Availability Statement

The raw data supporting the conclusions of this article will be made available by the authors, without undue reservation.

## Ethics Statement 

Ethical review and approval was not required for the study on human participants in accordance with the local legislation and institutional requirements. The patients/participants provided their written informed consent to participate in this study. Written informed consent was obtained from the individual(s) for the publication of any potentially identifiable images or data included in this article.

## Author Contributions

Study conception and design: SD’A and CEA. Data acquisition: EB, SD’A, CK, RS, CF, CRA, and CEA. Analysis and interpretation of data: EB, SD’A, CK, RS, CF, CRA, and CEA. Drafting or critical revision of manuscript: EB, SD’A, CK, RS, CF, CRA, and CEA. All authors contributed to the article and approved the submitted version.

## Funding

This study was supported in part by Cancer Center Support Grant (NIH/NCI P30 CA008748) and by P50 CA217694 (CRA). The funders had no role in the design and conduct of the study; collection, management, analysis, and interpretation of the data; preparation, review, or approval of the manuscript; and decision to submit the manuscript for publication.

## Conflict of Interest

CK reports receiving research funding from Merck, Amgen, and Agios; SD’A reports research funding from Amgen, EMD Serono, Incyte, Merck & Co., Nektar, Bristol-Myers Squibb, and Deciphera; consultancy for Amgen, EMD Serono, GlaxoSmithKline, Immune Design, Incyte, Merck & Co., Nektar, and Immunocore; reimbursement for travel expenses from EMD Serono, Merck & Co., Adaptimmune, and Immunocore.

The remaining authors declare that the research was conducted in the absence of any commercial or financial relationships that could be construed as a potential conflict of interest.

## Publisher’s Note

All claims expressed in this article are solely those of the authors and do not necessarily represent those of their affiliated organizations, or those of the publisher, the editors and the reviewers. Any product that may be evaluated in this article, or claim that may be made by its manufacturer, is not guaranteed or endorsed by the publisher.
